# Relations of drug use and socioeconomic factors with adherence to dental treatment among adolescents

**DOI:** 10.1186/s12903-018-0674-4

**Published:** 2018-12-19

**Authors:** Sílvia Letícia Freddo, Inara Pereira da Cunha, Jaqueline Vilela Bulgareli, Yuri Wanderley Cavalcanti, Antonio Carlos Pereira

**Affiliations:** 1 Department of Community Dentistry, Piracicaba Dental School, State University of Campinas, Av. Limeira, 901, P.O. BOX 52, Piracicaba, SP 13414-903 Brazil; 2 Department of Community Dentistry, Piracicaba Dental School, State University of Campinas, Av. Limeira, 901, P.O. BOX 52, Piracicaba, SP 13414-903 Brazil; 3 Department of Clinical and Social Dentistry, Federal University of Paraíba, João Pessoa, Brazil

**Keywords:** Adhesion, Illicit drugs, Oral health

## Abstract

**Background:**

Adolescents are vulnerable to behaviors that weaken health, by adopting habits that interfere with adherence to treatment. The aims of the present study were to investigate adolescents’ adherence to dental treatment and the relations between this behavior and socioeconomic factors and consumption of licit and illicit chemical substances.

**Methods:**

A longitudinal study was conducted with 474 adolescents from Piracicaba/SP/Brazil, who initially underwent a dental examination to verify the adherence for dental treatment. After 18 months, 325 adolescents were reassessed. Valid questions about socioeconomic conditions and use of alcohol and drugs were applied to participants. The chi-square test and Fisher’s exact test were used. The prevalence ratios were estimated with the respective 95% confidence intervals, using generalized linear models with Poisson distribution.

**Results:**

Eighteen (18) months after the first consultation, 325 adolescents were reassessed: 161 (49%) did not adhere to the treatment, and 164 (51%) adhered to it and answered the socioeconomic and alcohol and illicit drug questionnaires. Their mean age was 15 ± 1 years; of them, 189 (58%) were female. The prevalence of adherence to treatment decreased in patients without their own home (*p* = 0.034). In the individual analysis of the variables, drinking alcohol alone, experimenting with drugs, and proximity of friends who consumed illicit substances were associated with the outcome (*p* < 0.05). However, in the joint analysis, only proximity of friends who consumed drugs was the factor related to low adherence to dental treatment among the adolescents (*p* = 0.035).

**Conclusion:**

Adolescents who consumed alcohol and socialized with friends who used illicit drugs had greater difficulty in adhering to dental treatment.

## Background

According to data from the World Drug Report, the most commonly used illicit drugs are: marijuana, amphetamines, opioids and cocaine, respectively. The type of drug used in the world is not uniform. In America, the most commonly used drug is cocaine, while in Europe and Asia the most common types used are opioids [1].

Illicit drugs are prohibited by law. Legalized drugs, produced, consumed and marketed without restrictions are considered licit. Among the licit types, those most consumed by the Brazilian population are alcohol and tobacco [2]. The WHO has warned that the fact that there is no legislative ban on licit drugs makes them dangerous. While illicit drugs account for 0.8% of global health problems, alcohol and tobacco together account for 8.1% of the world’s health problems [3].

Through appeals made by the media, they encourage the consumption of alcohol and cigarettes among adolescents, transforming this incentive of consumption into a rite of passage into adulthood [4].

In Brazil, approximately 21 million boys and girls experience a period of development and maturation both physical and psychological, known as adolescence [5]. This is the phase of construction of the individual’s autonomy, a phase of adopting practices that were previously determined mainly by parents or guardians [6]. In this scenario of transformations, adolescents may become more vulnerable to behaviors that weaken health. Where oral health is concerned, eating habits and neglect of oral hygiene are preponderant factors for the establishment of oral diseases [7, 8].

Despite extensive coverage and investment, making use of health services results from of the interaction of multiple factors [9]. Thus, merely offering the services does not guarantee their use and access to them by the population [10, 11]. Among the factors involved in the use of dental services are social disparities, economic conditions and educational level [12, 13].

Non-adherence to treatment, due to its magnitude, is a public health problem, as it is related to the involvement and aggravation of oral diseases, negatively affecting adolescents’ quality of life [14, 15]. The present study adopted the concept of “adherence”, as the decision to seek a health service and finish the recommended treatment. Attitudes opposed to this idea were considered non-adherence [16].

The adherence to treatment may also be related to socioeconomic factors and cultural habits. Among adults, the consumption of alcohol and illicit drugs impairs the acceptance of medical recommendations [17]. In the adolescence, regular consumption of licit substances causes less demand for preventive services [18]. However, there is a scarcity of studies of this behavior among adolescents with a focus on adherence to dental treatment, especially with a longitudinal approach, showing evidence of the novelty of the theme.

Therefore, the aims of this study were to investigate adolescents’ adherence to dental treatment and the relations of this behavior with socioeconomic factors and consumption of licit and illicit chemical substances.

## Method

This research was approved by the Research Ethics Committee of Unicamp, Protocol no. 027/2011, in accordance with Resolution 466/12 of the National Health Council, concerning research with human beings. The Term of Free and Informed Consent (TFIC) was signed by those responsible for the adolescents.

### Initial phase

This was a longitudinal analytical study whose target population was adolescents from 15 to 19 years old living in Piracicaba, who attended the health units in which they were registered in the year 2015.

The municipality of Piracicaba has a population of 391,449 inhabitants, in which 28,539 adolescents live in the municipality. The sample size was calculated based on previous studies [19, 20]. The sample of baseline study was based on the caries experience in the Southeastern region of Brazil, using data from a previous national epidemiological survey, considering a sampling error of 5%, DMFT = 5.16 with SD = 4.54, sample loss of 20% and a level of confidence 95%, thus obtaining a sample of 1428 individuals aged 15 to 19 years, proportionally and randomly taken from 34 Primary Health Care Units (PHCU) in areas existent in the municipality. Of these 1428 adolescents initially selected, 249 failed to appear on the day of the exam or did not wish to participate. Thus, 1179 adolescents were examined. The majority of them had lived in the same suburb since their birth.

The inclusion criteria were to have participated in the baseline study and to be enrolled in the PHCU.

### Final phase (study of adherence)

Prior to this research, the oral health and quality of life of 1179 adolescents were evaluated in Baseline. Of these, 474 were referred for dental treatment. After 18 months, 325 adolescents were reassessed. The loss of 149 individuals, equivalent to 31.5% of the total sample, was due to: change of address or contact telephone number – 131 (88%); Transfer to another municipality – 9 (1%) and refusal of new participation – 9 (1%). Nevertheless, authors consider data was not biased due to the participants withdraw. No statistically significant differences were detected between the socioeconomic data of all initially enrolled participants (*n* = 474) and those maintained at the follow up (*n* = 325).

**Fig. 1:**
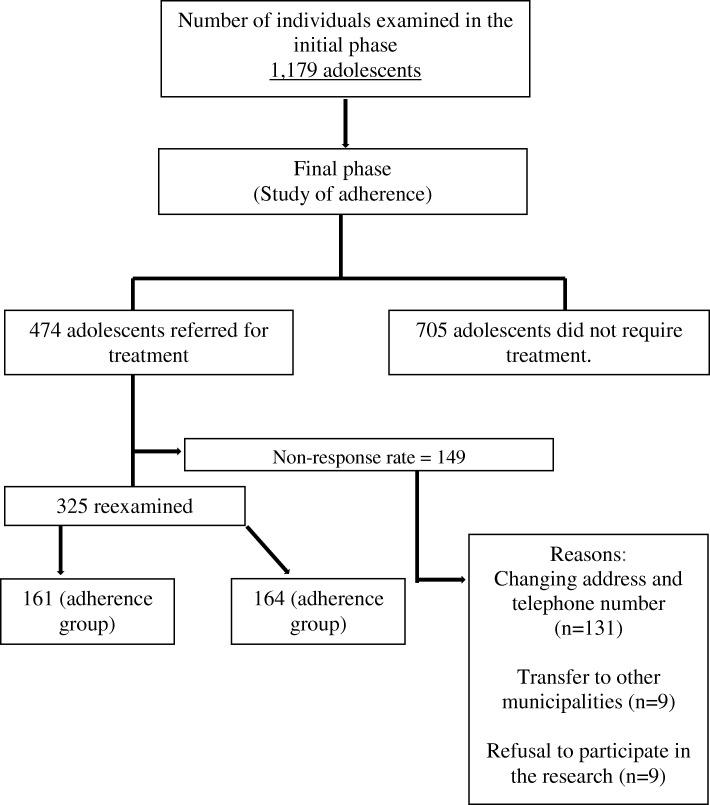
Representative flowchart of the study phases (Bulgareli, 2016) [21]

For better visualization of this study design, Fig. 1 (Bulgareli, 2016) [21] explains the sequence of the developmental stages of the study.

About 18 months after Baseline, the researchers conducted an active search to locate the adolescents who were referred for dental treatment in the baseline.

The data collected in the final phase of the study were adherence to dental treatment and experience of alcohol and illicit drugs consume. The dental tratament adherence was considered as the decision to seek a health service and finish the recommended treatment. The clinical examination was carried out to verify those individuals with absence of caries and peridontal disease that is adherence to dental treatment.

The process of calibrating the two examiners for the clinical conditions was conducted by a Gold Standard examiner, and followed a similar methodology from a previously published study [20]. Theoretical-practical activities were performed: 1 theoretical (4 h), 4 clinical training sessions (4 h each), 2 calibration exercises (4 h each). The training stage consisted of a theoretical discussion, followed by a practical stage, in which the examiners evaluated 12 adolescents per period. The final calibration exercise consisted of 2 periods (total of 8 h) with mean inter-examiner Kappa values of 0.95. For the purpose of verifying maintenance of the diagnostic criteria and intra-examiner error, 10% of the sample were re-examined, showing a mean Kappa value of 0.96 for all the clinical conditions.

### Study variables

Data were collected by previously trained, calibrated examiners, using four self-administered questionnaires and clinical dental examination to verify adherence. The first questionnaire developed for the purposes of this study provided data regarding adherence to dental treatment, considering the outcome of the study. It was elaborated by the researcher of the study. The second questionnaire was developed the World Health Organization [22] and provided data on use (experience) of alcohol and illicit drugs. In the baseline the collected variables were socioeconomic characterization (family income, father’s and mother’s literacy, type of housing) [23] and data on adolescents’ school failure and their insertion into the labor market obtained by means of the Goes questionnaire (2001) [24].

**Fig. 2:**
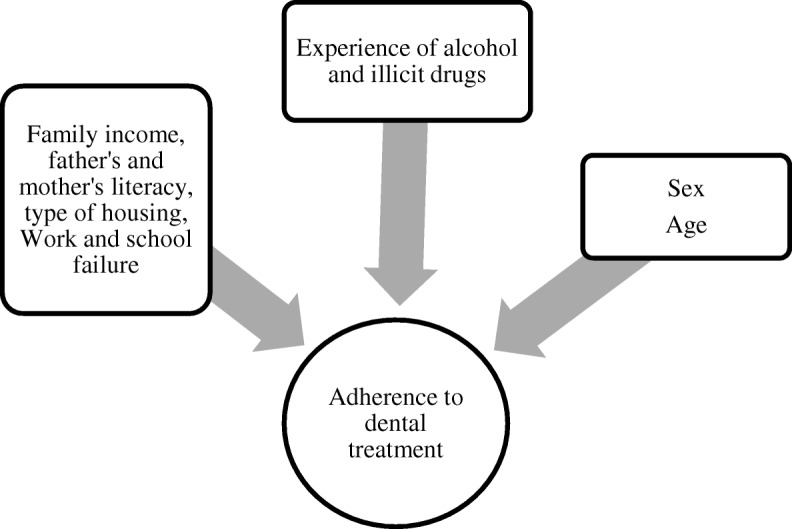
Theoretical Model for Study of Association between Independent Variables and Adherence to Dental Treatment

The dependent variable of the study was adherence to dental treatment and the independent types were use (experience) of alcohol and illicit drugs, both collected in the final phase and socioeconomic factors (family income, father’s and mother’s literacy, type of housing, Work and school failure). The adolescents’ sex and age were also considered independent variables collected in the final phase (Fig. 2).

### Data analysis

In the data analysis, the quantitative characteristics were described according to adherence by using mean and standard deviation and the groups compared according to adherence using the Student’s-*t* test. The qualitative characteristics evaluated were described according to adherence with the use of absolute and relative frequencies. In addition, Chi-square and Fisher’s exact tests or likelihood ratio tests were used to verify whether there was association with adherence [25].

The prevalence ratios were estimated with the respective 95% confidence intervals, using generalized linear models with Poisson distribution and logarithmic link function for each parameter evaluated [26]. All the prevalence values were adjusted by age. The joint model of the characteristics evaluated was estimated to explain the adherence to treatment with the variables that presented a descriptive level lower than 0.2 (*p* < 0.2) in the bivariate tests, by using the same analysis methodology.

The analyses were performed using IBM-SPSS for Windows software version 20.0 and the tables were elaborated using Microsoft-Excel 2003 software. The tests were performed with significance level of 5%.

## Results

A total of 325 adolescents were evaluated; of these, 189 (58%) were female and 136 (42%) were male. Of the total sample, 164 (51%) adolescents adhered to treatment and 161 (49%) did not. Among those who adhered, 96 (58%) were female and 68 (42%) male. The mean age of the total sample was 15 ± 1 years.

Relative to the socioeconomic data of the families of the 164 adherents, 46 (29%) adolescents had income higher than 1 or up to 2 minimum wages. As regards their parents, 49 (32.5%) had a literacy level of up to 2nd grade incomplete, a value lower than that of the mother’s literacy level. The type of dwelling that prevailed was their own residence, 125 (77.6%). Considering school achievement and insertion in the labor market, 131 (81%) never failed in school and 134 (82%) did not work (Table 1). There was no statistically significant difference between the family income of the adolescents and the outcome.

**Table 1:** Description of the personal characteristics of the adolescents according to adherence and results of the statistical tests

Variable	Total (N = 325)	Adherence		CI (95%)		
		Yes (*N* = 164)	PR	Lower	Upper	*p*
Sex, n (%)						0.962
Female	189 (58.2)	96 (58.3)	1.00			
Male	136 (41.8)	68 (41.7)	1.00	0.80	1.24	
Age (years)			0.91	0.81	1.03	0.122^b^
mean ± SD	15.2 ± 1	15.1 ± 0.94				
Income, n (%)						0.742^a^
1 MW	26 (8.2)	12 (7.5)	1.00			
+ 1 up to 2 MW	102 (32)	46 (28.7)	0.98	0.61	1.56	
+ 2 up to 3 MW	77 (24.1)	38 (23.8)	1.07	0.67	1.72	
+ 3 up to 5 MW	69 (21.6)	38 (23.8)	1.19	0.75	1.90	
+ 5 up to 7 MW	19 (6)	10 (6.2)	1.14	0.63	2.07	
+ 7 up to 10.5 MW	17 (5.3)	11 (6.9)	1.40	0.81	2.42	
+ 10.5 MW	9 (2.8)	5 (3.1)	1.20	0.59	2.46	
Father’s education, n (%)						0.239
Illiterate up to some 4th grade incomplete	22 (7.4)	7 (4.6)	1.00			
4th grade complete or 5th to 8th grade incomplete	84 (28.1)	49 (32.5)	1.83	0.97	3.47	
8th grade complete or high school incomplete	96 (32.1)	49 (32.5)	1.60	0.84	3.05	
High school or incomplete college	78 (26.1)	37 (24.5)	1.49	0.77	2.87	
College degree	19 (6.4)	9 (6)	1.49	0.69	3.23	
Mother’s education (%)						0.167
Illiterate up to some 4th grade incomplete	16 (5)	7 (4.3)	1.00			
4th grade complete or 5th to 8th grade incomplete	71 (22.2)	28 (17.4)	0.90	0.48	1.69	
8th grade complete or high school incomplete	122 (38.1)	70 (43.5)	1.31	0.74	2.33	
High school or incomplete college	86 (26.9)	42 (26.1)	1.12	0.62	2.03	
College degree	25 (7.8)	14 (8.7)	1.28	0.66	2.46	
Housing, n (%)						0.034
Owner	233 (72.4)	125 (77.6)	1.00			
Not owner	89 (27.6)	36 (22.4)	0.75	0.57	1.00	
School Failure, n (%)						0.413
No	256 (79)	131 (80.9)	1.00			
Yes	68 (21)	31 (19.1)	0.89	0.67	1.19	
Employment, n (%)						0.466
No	262 (80.6)	134 (82.2)	1.00			
Yes	63 (19.4)	29 (17.8)	0.90	0.67	1.21	

Source: Elaborated by the author based on data from the city of Piracicaba

*MW* Abbreviation for minimum wage. Chi-square test; ^a^ Probability ratio test; ^b^ Student’s*-t* test; *PR* Prevalence Ratio, *CI* Confidence Interval; Not all patients had all the information; & this could not be estimated

Table 1 - from the personal characteristics evaluated, in isolation - shows only the type of housing had a statistically significant influence on adherence to treatment (*p* = 0.034), and the prevalence of adherence to treatment decreased in patients without their own home.

As regards alcohol consumption, 105 participants (64%) reported having previously tried some type of alcoholic drink, and 30 adolescents (18.5%) used it for the first time, in the home. When asked about alcohol consumption in the last 30 days, 41 (25%) reported having consumed it; 16 (10%) answered that they had problems due to alcohol, and 10 (6%) had problems once or twice in that period. Relative to alcohol abuse, 37 (23%) of the adolescents reported having become really drunk, and for 31 of them (19%), the frequency was one to two times.

When asked who they used to drink with, 57 (35%) drank with their friends. In the comparison between groups, the variables “tried alcohol” (*p* = 0.035) and “drinking alone” (*p* = 0.029) were statistically significant.

As regards drug use, 155 (96.9%) of the adolescents had never tried drugs; only four of them (2.5%) reported having used drugs in the last 30 days; and three (1.8%) of them reported the frequency as being once or twice. With reference to their circle of friends, 56 (34.4%) reported that their friends did some type of drug. There was a significant association between nonadherence to dental treatment and having friends who did drugs (*p* = 0.005) and the variable relative to those who experienced drugs was also statistically significant (*p* = 0.049).

The factors having experienced alcohol, drinking alone, having tried drugs and having friends who did drugs had a single influence on the prevalence of adherence to dental treatment (*p* < 0.05), all of which decreased the prevalence of adherence to treatment when these parameters were present (Table 2).

**Table 2:** Description of alcohol and drug consumption characteristics of the adolescents according to adherence and results of statistical tests

Variable	Total (N = 325)	Adherence			CI (95%)	
		Yes (N = 164)	RP	Lower	Upper	*p*
Tried alcohol, n (%)						0.035
No	98 (30.2)	58 (35.6)	1.00			
Yes	226 (69.8)	06 (64.4)	0.79	0.63	0.97	
Age when you tried alcohol			1.06	0.98	1.14	0.155^c^
mean ± SD	14.4 ± 1.97	14.6 ± 1.98				
Where alcohol was used						
House, n (%)						0.453
Yes	257 (79.8)	132 (81.5)	1.00			
Own home or friend’s home, n (%)						0.434
Yes	57 (17.7)	26 (16)	0.89	0.65	1.21	
School, n (%)						0.497^a^
Yes	1 (0.3)	0 (0)	&			
Street or park, n (%)						0.554
Yes	25 (7.8)	14 (8.6)	1.12	0.78	1.62	
Bar or club, n (%)						0.718
Yes	28 (8.7)	15 (9.3)	1.07	0.75	1.54	
Restaurants, n (%)						0.722^a^
Yes	7 (2.2)	3 (1.9)	0.85	0.36	2.01	
Elsewhere, n (%)						0.155
Yes	51 (15.8)	21 (13)	0.79	0.56	1.12	
How many times did you drink alcohol in the last 30 days, n (%)						0.220^b^
None	226 (69.8)	122 (74.8)	1.00			
1 to 2 times	66 (20.4)	29 (17.8)	0.81	0.60	1.10	
3 to 9 times	24 (7.4)	9 (5.5)	0.69	0.41	1.18	
+ 10 times	8 (2.5)	3 (1.8)	0.69	0.28	1.71	
How many times did you have alcohol problems in the last 30 days, n (%)						0.262^b^
None	293 (90.4)	147 (90.2)	1.00			
1 to 2 times	21 (6.5)	10 (6.1)	0.95	0.60	1.51	
3 to 9 times	6 (1.9)	5 (3.1)	1.66	1.14	2.42	
+ 10 times	4 (1.2)	1 (0.6)	0.50	0.09	2.73	
In your life, how many times have you been drunk, n (%)						0.301^b^
None	238 (73.5)	126 (77.3)	1.00			
1 to 2 times	71 (21.9)	31 (19)	0.82	0.62	1.10	
3 to 9 times	12 (3.7)	4 (2.5)	0.63	0.28	1.41	
+ 10 times	3 (0.9)	2 (1.2)	1.26	0.56	2.83	
If you came home drunk, how would your family react, n (%)						0.388
Would not notice	28 (8.7)	14 (8.6)	1.00			
Would notice. But would not care	14 (4.3)	4 (2.5)	0.57	0.23	1.42	
Would notice and would get upset	54 (16.7)	31 (19.1)	1.15	0.74	1.78	
Would notice and would get very upset	155 (48)	75 (46.3)	0.97	0.65	1.45	
Do not know	72 (22.3)	38 (23.5)	1.06	0.69	1.62	
Do your parents drink, n (%)						0.809^b^
No	149 (46)	73 (44.8)	1.00			
Father	100 (30.9)	48 (29.4)	0.98	0.75	1.27	
Mother	22 (6.8)	13 (8)	1.21	0.82	1.77	
Both	50 (15.4)	27 (16.6)	1.10	0.81	1.49	
Do not know	3 (0.9)	2 (1.2)	1.36	0.60	3.08	
Drink alcohol, n (%)						0.222
No	164 (50.6)	88 (54)	1.00			
Yes	160 (49.4)	75 (46)	0.87	0.70	1.09	
Drink with friends, n (%)						0.145
No	198 (61.1)	106 (65)	1.00			
Yes	126 (38.9)	57 (35)	0.85	0.67	1.07	
Drink with family members, n (%)						0.746
No	290 (89.5)	145 (89)	1.00			
Yes	34 (10.5)	18 (11)	1.06	0.76	1.48	
Drink with other people, n (%)						> 0.999^a^
No	317 (97.8)	159 (97.5)	1.00			
Yes	7 (2.2)	4 (2.5)	1.14	0.59	2.18	
Drink alone, n (%)						0.029^a^
No	319 (98.5)	163 (100)	1.00			
Yes	5 (1.5)	0 (0)	&			
Tried drugs, n (%)						0.049
No	306 (94.4)	158 (96.9)	1.00			
Yes	18 (5.6)	5 (3.1)	0.54	0.25	1.14	
Age when tried drugs			0.81	0.52	1.25	0.426^c^
média ± SD	14.8 ± 1.38	14.4 ± 1.52				
Did any type of drugs in the past 90 days, n (%)						0.540^a^
No	314 (96.9)	159 (97.5)	1.00			
Yes	10 (3.1)	4 (2.5)	0.79	0.37	1.70	
How many times have used any drugs in the past 30 days, n (%)						0.445^b^
None	314 (96.9)	159 (97.5)	1.00			
1 to 2 times	5 (1.5)	3 (1.8)	1.19	0.57	2.44	
3 to 9 times	1 (0.3)	0 (0)	&			
+ 10 times	4 (1.2)	1 (0.6)	0.49	0.09	2.70	
Your friends do drugs, n (%)						0.005
No	188 (58)	107 (65.6)	1.00			
Yes	136 (42)	56 (34.4)	0.72	0.57	0.92	
You do drugs, n (%)						0.347
No	313 (96.6)	159 (97.5)	1.00			
Yes	11 (3.4)	4 (2.5)	0.72	0.32	1.58	

Source: Elaborated by the author based on data from the city of Piracicaba.Chi-square test; ^a^ Fisher exact test; ^b^ Probability ratio test; ^c^ Student’s t-test; *PR* Prevalence Ratio, *CI* Confidence Interval; Not all patients have all the information; & it cannot be estimated

Table 3 shows that only the fact of having friends who did drugs influenced the prevalence of adherence to dental treatment (*p* = 0.035); and the prevalence of adherence decreased by 23% in patients with friends who did drugs.

**Table 3:** Result of the joint model to explain adherence to treatment of the adolescents

Variable	PR	CI (95%)		*p*
		Inferior	Superior	
Age (years)	0.92	0.82	1.04	0.189
Housing				
Owner	1.00			
Not owner	0.80	0.60	1.05	0.109
Tried alcohol				
No	1.00			
Yes	0.86	0.69	1.07	0.167
Friends do drugs				
No	1.00			
Yes	0.77	0.60	0.98	0.035

Source: Elaborated by the author based on data from the city of Piracicaba

*PR* Prevalence Ratio, *CI* Confidence Interval

## Discussion

The present study revealed that low adherence to dental treatment among adolescents was associated in isolation with socioeconomic factors (type of housing) and behavioral factors such as alcohol consumption and friendships. However, in the joint analysis of the variables, proximity of friends who used illicit drugs was revealed to be the significant factor for lower adherence to treatment.

The variable ‘type of housing,’ when tested alone was statistically significant with the outcome, the use of dental services was associated with socioeconomic factors, among others. In a nationwide study, the researchers found that the number of people who did not have access to dental health services was 16 times higher among those in need, and that this population also had the greatest difficulty in receiving care when they sought the service. In addition to the lack of care to meet the demand, another factor was the perception that people had about the quality of care provided by public health services, leading to the population seeking private services [27].

According to the WHO [28], there are six dimensions involved in adherence to treatment: individual characteristics of the patient (gender, age, ethnicity, marital status, educational level and socioeconomic level), presence of the disease (chronicity, symptoms and consequences), cultural habits (perception of disease, beliefs, knowledge on the problem), aspects related to the treatment (cost, effects, therapeutic protocols), institution (public health policy, organization of services) and relationship with the health team [29].

Therefore, subjects do not adhere to treatment by their will alone, as is the common opinion, but adherence is structured in a multidimensional manner [30]. In childhood, for example, family behavior influences children’s access to dental appointments [31]. Thus, the use of services is low in schoolchildren whose mothers have low schooling and worse economic conditions [32, 33].

However, a qualitative study with adolescents found that among the reasons for non-adherence to dental treatment were the absence of priority and neglect of care. This was because the need to attend the consultation probably had no meaning in the context of adolescents, because it was of less importance in their daily lives [20]. According to Leão et al. (2015) [34], adolescents accessed the service mainly for curative treatment, and sought care due to toothache or esthetics issues [35]. These findings supported the fact that socioeconomic factors were less important for the outcome in the study. In this study, the issue of adherence was individual and not programmatic, since adolescents did not have problems with access to health services.

Consumption of alcohol and illicit drug use among adolescents is a worldwide concern [36]. Despite the Child and Adolescent Statute (ECA) [37] having classified the sale, supply and delivery of substances capable of causing dependence in children or adolescents as criminal actions, it has been observed that these (criminal) actions were frequently practiced in daily life. Both the permissiveness of families and society as well as the lack of supervision contribute to drug experimentation among children and adolescents [36].

Consequently, a research in a Brazilian state revealed the high prevalence of alcohol consumption (24%) and other drugs (2.3%) in 8th graders [38]. The percentages were higher in the present study, considering the studied age range and the methodological approach.

Most adolescents first experienced alcohol at home – 65 (20%), a finding similar to that in the literature [39, 40]. Thus, health education strategies for this population should consider a family approach, since the family can be a facilitator of access to and consumption of alcohol. To achieve this, inter-departmental actions, involving health and community need to be elaborated, or even including this approach in education, as suggested in the proposal developed by the Health in School Program (PSE) [41].

The motivation for alcohol use among adolescents was in the narcotic effect and socialization with their peers, whereas the search for illicit drugs referred to the escape from personal and family problems [42]. The illicit drugs had a lower prevalence of consumption (4%) when compared with alcohol (46%), corroborating results of other Brazilian studies [42, 43].

Important to emphasize is the fact that the use of drugs by friends was associated with non-adherence to dental treatment. This reflected the effect of the influence of the friends on the adoption of health care [41, 42]. Important to point out is that the use of illicit and licit substances may not be a socially acceptable behavior, and may lead to respondents omitting the fact of their use in questionnaires, even when these are self-applied. There was a loss of 149 individuals, equivalent to 31.5% of the total sample, was due to: change of address or contact telephone number – 131 (88%); Transfer to another municipality – 9 (1%) and refusal of new participation – 9 (1%).

In Brazil, the National Health System (SUS) is a public entity and offers the population services of low, medium and high complexity. Low complexity, or Primary Care, coordinates the action of health prevention and promotion, elaborated and provided in a multi-professional manner by the Family Health Teams. Within the FHS, there are the Oral Health Teams (ESB), responsible for dental assistance and preventive care for a certain number of families [44]. Therefore, the adolescents who participated in the present research, received dental attendance by SUS, thus, the results obtained may contribute to the strategies of promotion, prevention and adherence to dental treatment by those individual who use the public system. The ESB needs to increasingly observe the relations existent between patients and the environment in which they live, including their family and friendship relationships, particularly where the care of adolescents is concerned.

The main limitation the study, of course, is related to the non-response rate, since we had difficulty locating important part of the sample of adolescents, although they have been sought in schools where they studied, in the PHCU, and also in their homes (there were often three attempts find them). However, as relevance of this study is due to the professional has the opportunity to understand the expectations and characteristics of individuals who do not follow the recommended treatment, which allows more individualized interventions to improve adherence and hence provide a more qualified service.

## Conclusion

Adolescents who consumed alcohol and socialized with friends who did illicit drugs had greater difficulty in adhering to dental treatment. Therefore, adherence to dental treatment was largely associated with individual behaviors and friendship relationships, rather than with socioeconomic factors.

## Abbreviations

ECA: Child and Adolescent Statute
ICF: The Informed Consent Form
SUS: Unified Health System
WHO: World Health Organization
